# Joint scatterer localization and material identification using radio access technology

**DOI:** 10.1186/s13638-022-02167-7

**Published:** 2022-09-17

**Authors:** Yi Geng, Deep Shrestha, Vijaya Yajnanarayana, Erik Dahlman, Ali Behravan

**Affiliations:** 1 Ericsson R &D, Nanjing, China; 2 Ericsson Research, Linköping, Sweden; 3 Ericsson Research, Bangalore, India; 4 Ericsson Research, Kista, Sweden

**Keywords:** Scatterer material identification, Scatterer localization, Reflection loss, Path loss, Incident angle, Joint communication and sensing

## Abstract

Cellular network technologies and radar sensing technologies have been developing in parallel for decades. Instead of developing two individual technologies, the 6G cellular network is expected to naturally support both communication and radar functionalities with shared hardware and carrier frequencies. In this regard, radio access technology (RAT)-based scatterer localization system is one of the important aspects of joint communication and sensing system that uses communication signals between transceivers to determine the location of scatterers in and around the propagation paths. In this article, we first identify the challenges of the RAT-based scatterer localization system and then present single- and multiple-bounce reflection loss simulation results for three common building materials in indoor environments. We also propose two novel methods to jointly localize and identify the type of the scatterers in a rich scattering environment.

## Introduction

Depending on whether the objects of interest have communication capability or not, the localization technologies are classified into active localization and passive localization [1]. Active localization systems use transmitted and received signals from both the localization system and objects with communication capability to localize the objects. Almost all RAT-based localization systems are active localization (e.g., localizing user equipment (UE) in a cellular network). Passive localization systems localize scatterers without communication capability by exploiting reflected signals induced by the scatterers (e.g., detecting a plane by using a bistatic radar).

Unlike the existing passive localization use cases, some emerging 6G localization applications such as robotic perception, virtual reality (VR), digital twins, and three-dimensional (3D) digital mapping require scatterer information of not only the position but also the material. By combining the scatterers’ location information with their material information of an environment, a 3D digital map with another layer of material information can be generated. This supplementary information in the 3D digital map can find applications in VR games and the emerging area of virtual tourism due to COVID-19-caused travel disruption. Current VR suits can give visual and audio illusions by simulating human senses of sight and hearing. However, with material information in the 3D digital map, VR applications can generate additional human sensations like touch and smell. For example, a VR game player can feel the hardness, the temperature, and the odor of the virtual objects in a simulated environment through sensory feedback. Material sensing can be a key requirement for autonomous driving (AD) also. Today, the onboard radar systems of vehicles are capable of estimating the precise position of obstacles around the vehicles. However, having added information about the type of material can aid in several ways. For example, by sensing the snow on the road, a vehicle can tune its onboard electronics such as the traction control system, or change to another safer route.

This article is organized as follows. In Sect. 2, we present the challenges of the RAT-based scatterer localization system. In Sect. 3, we analyze the RL of single- and double-bounce reflection for three common building materials in indoor environments. In Sect. 4, we propose two novel methods all based on RL to trace radio trajectories, localize scatterers’ position, and identify scatterers’ material in rich scattering environments. Section 5 discusses how the uncertainty in the proposed methods can be reduced. To the best of the authors’ knowledge, this is the first work that shows how to localize scatterers and identify scatterers’ material simultaneously by using wireless communication signals.

## Challenges

Detecting and localizing scatterers surrounding radio transceivers in the rich scattering environment is one of the emerging requirements of the JCAS system. Most scatterer localization technologies trace radio trajectories and localize objects using 3D digital maps, ray tracing methods, and geometrical optics theory [2]. These map-based localization methods can be implemented only when the corresponding maps of the environment are available to the localization systems [3]. Therefore, map-based scatterer localization methods not only use cellular infrastructure but also depend on imported 3D maps. However, the 3D map is not a component of conventional cellular networks. Moreover, environmental changes are likely to happen frequently, especially in indoor scenarios, and can lead to out-of-date information in the 3D digital maps. The objective of RAT-based scatterer localization is to sense and localize scatterers without requiring any external assistance beyond the scope of RAT capabilities, e.g., without importing the 3D digital map of an environment. With this restriction, tracing radio trajectory in a rich scattering environment is quite challenging, because radio signals can be reflected by scatterers at unknown positions and attenuated by an uncertain number of reflections. Possible information can be gleaned by the RAT-based scatterer localization system including

- Angle of departure (AOD);
- Angle of arrival (AOA);
- Time of flight (TOF);
- Transmit power and received signal strength (RSS);
- Positions of transmitters and receivers (anchor nodes).

AOD and AOA represent transmitter- and receiver-side beam direction, respectively. TOF can be used to estimate the total path length. RSS at the receiver side can be measured or calculated by link budget. Anchor nodes are transmitters or receivers with known coordinates [4].

In a rich scattering environment, non-line-of-sight (NLOS) radio signals typically undergo phenomena such as reflection, diffraction, and refraction. In this article, we only consider reflection for localization purposes. The reflection of NLOS trajectories can be either single- or multiple-bounce reflections. In prior works, the scatterer localization was done in a single-bounce context (single-bounce-assumption) [5–11]. Multiple-bounce reflection trajectories are excluded in most prior technologies to reduce the complexity of the localization algorithms. For example, the typical scatterer localization system “radar” detects scatterers by exploiting single-bounce reflection. The sparse scatterers (e.g., planes and missiles) in the air make the single-bounce-assumption reasonable for radar applications. Light detection and ranging (LiDAR) localizes scatterers with the laser by measuring the time for the single order reflection between the transceivers. However, such approaches may not apply to RAT-based scatterer localization systems deployed in rich scattering environments, in which a large number of multiple-bounce reflection paths exist, especially in urban areas or cluttered indoor scenarios. Radio signals in such environments are likely to be reflected multiple times. Therefore, single-bounce reflection may not be sufficient to characterize the sensing parameters of scatterers.

**Fig. 1:**
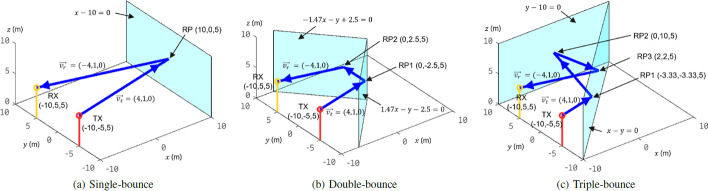
Trajectories of a radio ray between TX and RX with different numbers of reflections

With the single-bounce-assumption, the sensing reliability and accuracy of RAT-based scatterer localization degrade in the rich scattering environment. Scatterers may be mistakenly localized when radio rays are reflected between multiple scatterers. For example, as shown in Fig. 1, a TX antenna and a RX antenna are placed at ($-10, -5, 5$) and (− 10, 5, 5) in a Cartesian coordinate system, respectively. The TX transmits a narrow beam in the direction of vector $\overrightarrow{v_t}$ = (4, 1, 0), while the RX receives the beam in the direction of vector $\overrightarrow{v_r} = (-4, 1, 0)$. For this scenario, according to single-bounce assumption, the reflection point (RP) should locate at (10, 0, 5), the radio ray should be reflected by the plane $x-10=0$ producing single-bounce reflection, and the trajectory is described by point-to-point motion passing through the points TX($-10, -5, 5$), RP(10, 0, 5), and RX($-10$, 5, 5) as shown in Fig. 1a. Note that the scatterer represented by the plane $x-10=0$ should be a larger surface than the radius of the first Fresnel zone. To demonstrate the spatial relationship between the trajectory and the scatterers, the scatterer is plotted to an appreciable rectangle. However, the trajectory in Fig. 1a may not represent the true trajectory of the radio ray. For example, if two scatterers represented by the planes $1.47x-y-2.5=0$ and $-1.47x-y+2.5=0$ exist as shown in Fig. 1b, then the radio ray is reflected twice by the two scatterers and propagates along the trajectory passing through the points TX($-10, -5$, 5), RP1(0, $-2.5$, 5), RP2(0, 2.5, 5), and RX($-10$, 5, 5). If two scatterers $x-y=0$ and $y-10=0$ exist as shown in Fig. 1c, the radio ray is reflected three times and propagates along the points TX($-10, -5$, 5), RP1($-3.33, -3.33, 5$), RP2(0, 10, 5), RP3(2, 2, 5), and RX($-10$, 5, 5). Any RAT-based scatterer localization system based on the single-bounce assumption cannot distinguish the trajectories in Fig. 1a–c due to the same TX/RX locations, AOD, and AOA from the transceivers’ point of view. In rich scattering environments (e.g., the urban canyons or the indoor scenarios), there are abundant scatterers in and around the propagation paths; such multiple-bounce-dominant paths are mistakenly identified as single-bounce reflection by the single-bounce-assumption-based localization methods. For example, as shown in Fig. 1a, b, we assume that the sensors of an AD vehicle locate at ($-10, -5$, 5) and ($-10$, 5, 5), and two close obstacles (e.g., pedestrians or other vehicles) locate at (0, $-2.5$, 5) and (0, 2.5, 5). A single-bounce-assumption-based scatterer localization system cannot identify the two obstacles correctly: the two obstacles are identified as a single obstacle with the wrong location (10, 0, 5) in the distance. This error may lead to wrong driving decisions and fatal accidents for AD.

**Fig. 2:**
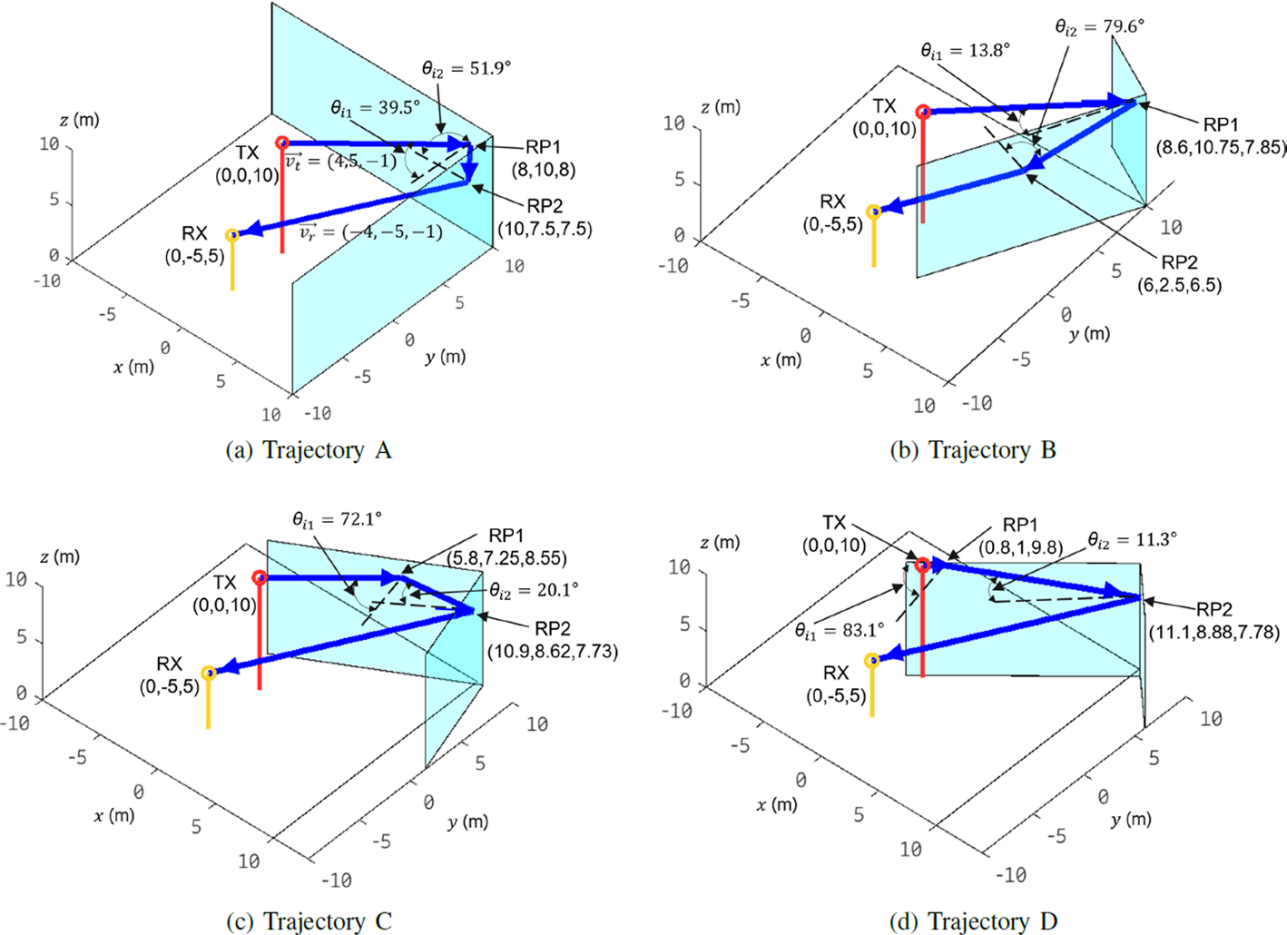
Four different scatterer layouts induce different trajectories of a radio ray

**Fig. 3:**
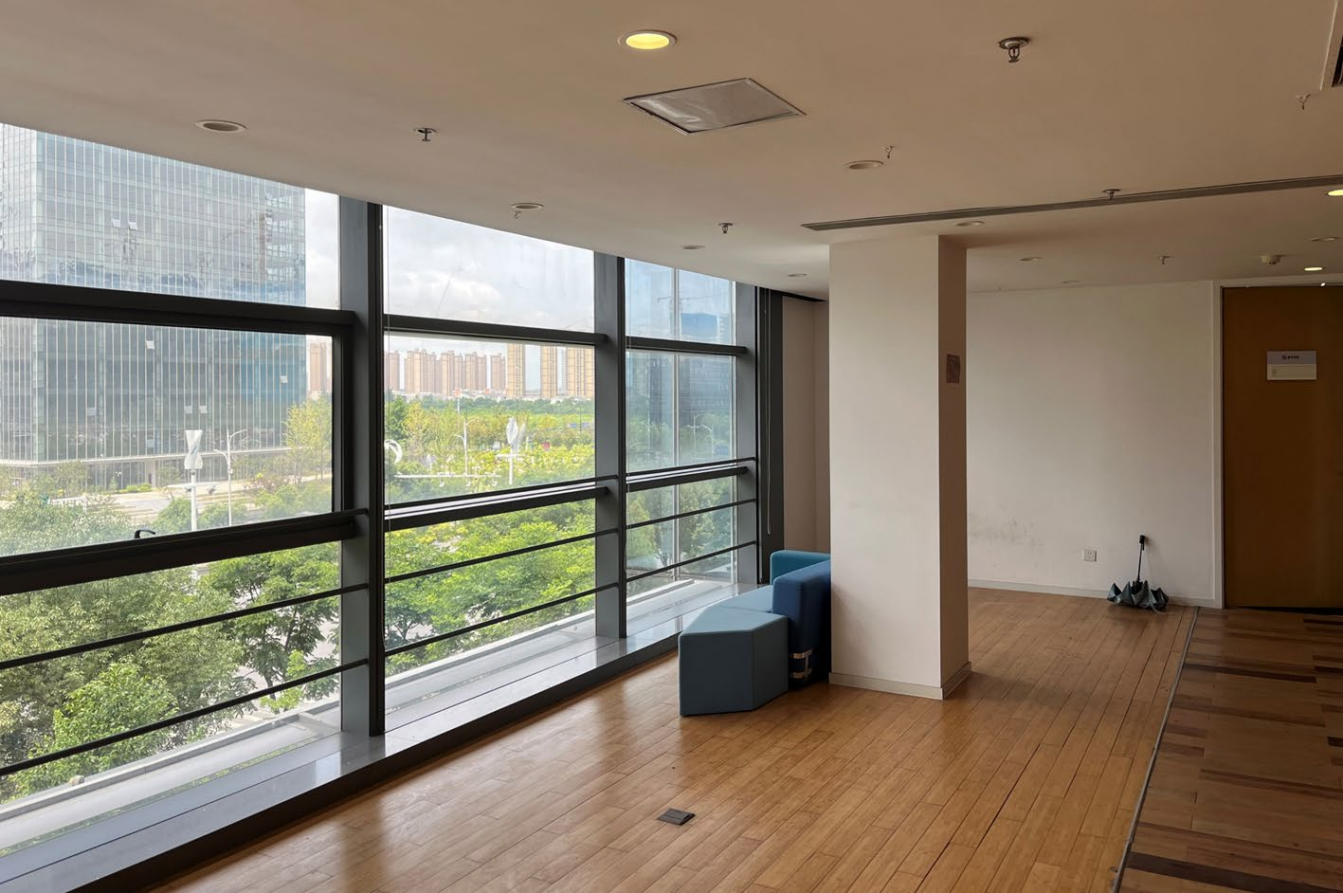
An exemplary indoor environment with wood, plaster, and glass

**Fig. 4:**
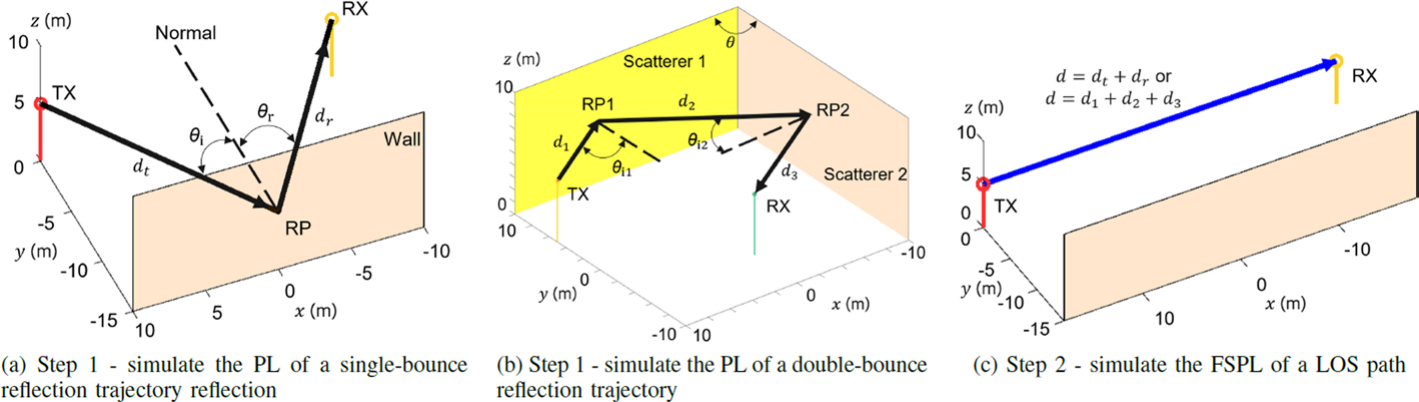
MATLAB-based simulation steps to get the RL of single- and double-bounce reflection

**Fig. 5:**
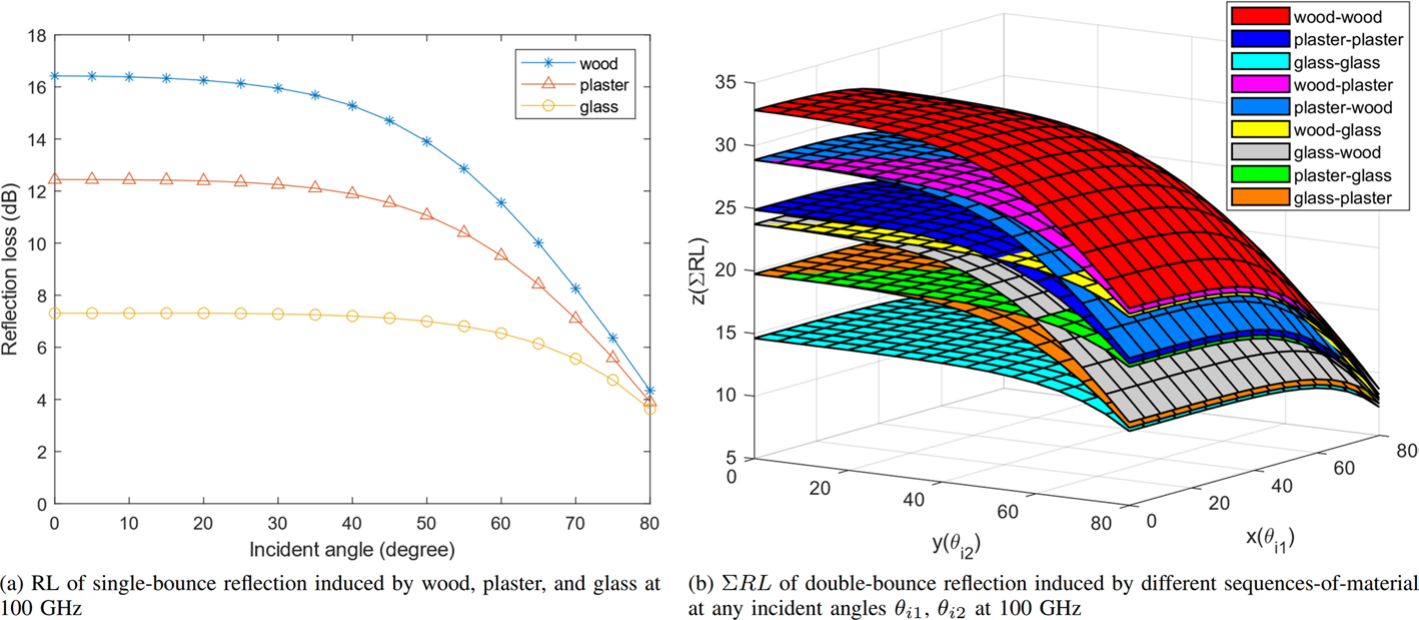
Simulated RL of single- and double-bounce reflection induced by wood, plaster, and glass

**Fig. 6:**
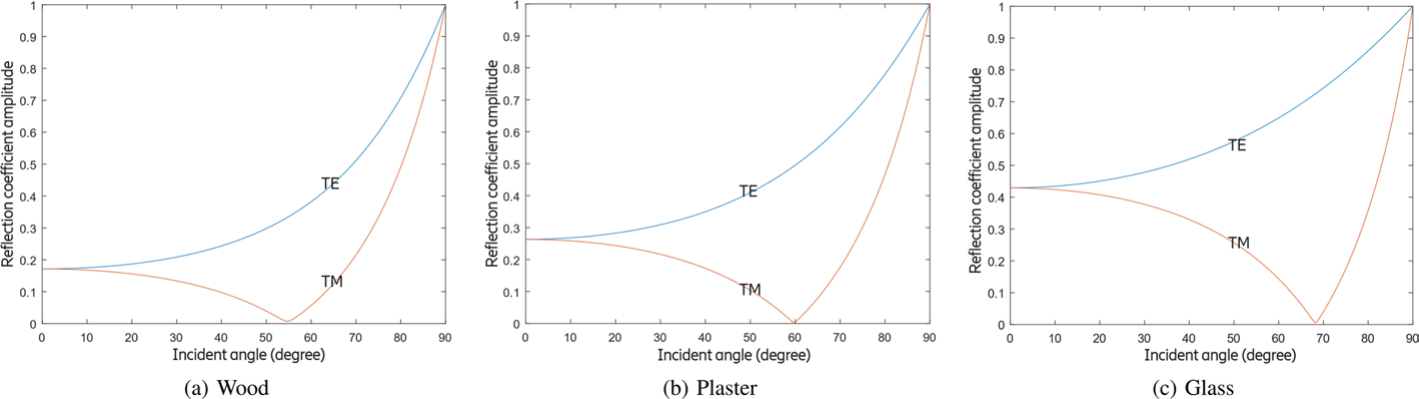
Reflection coefficient amplitudes for air/wood, air/plaster, and air/glass interfaces at 100 GHz

**Fig. 7:**
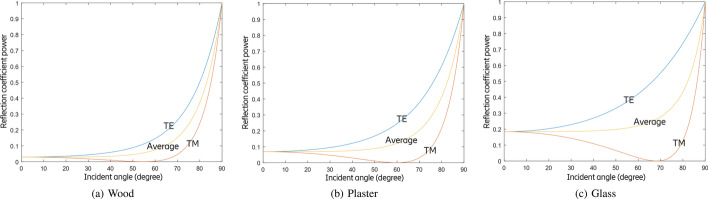
Reflection coefficient power for air/wood, air/plaster, and air/glass interfaces at 100 GHz

**Fig. 8:**
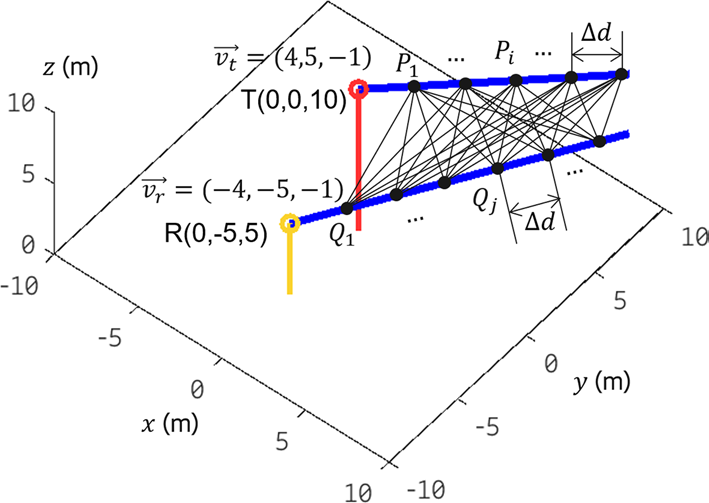
An illustration of the way the proposed method works

**Fig. 9:**
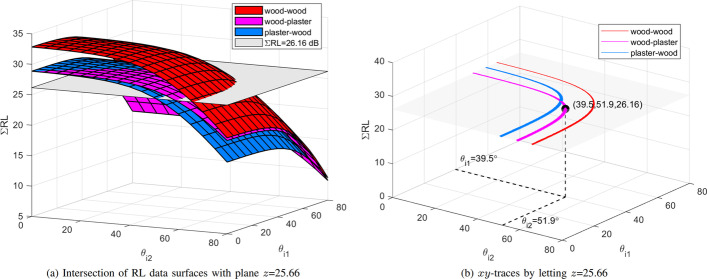
All possible sequences-of-material and incident angles that induce RL of 26.16 dB

**Fig. 10:**
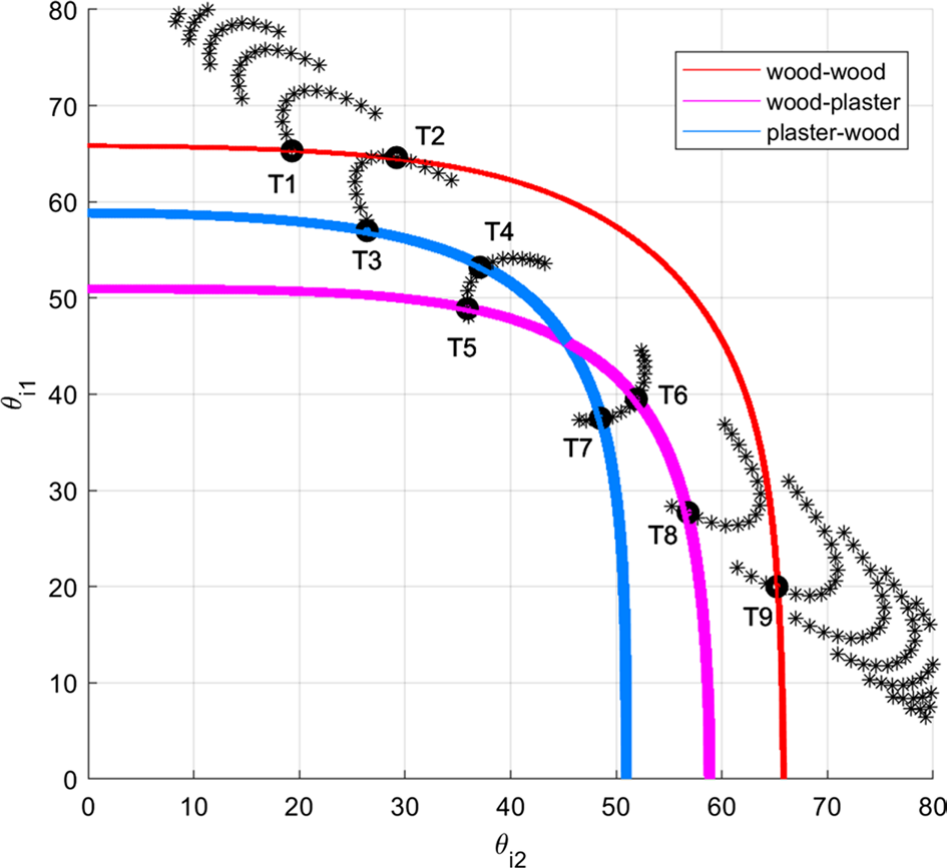
How the true trajectory is identified by Step 4 of method 2

**Fig. 11:**
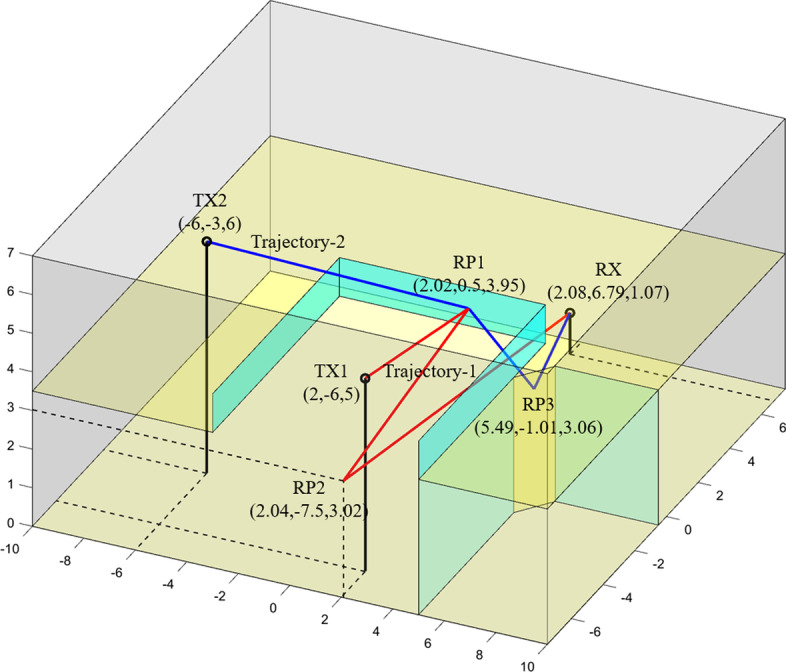
Two trajectories between TX1-RX and TX2-RX in an indoor environment

Even if we assume that an RAT-based scatterer localization system knows the number of bounces of the radio rays, we still cannot precisely trace the trajectories and localize the scatterers. For example, as shown in Fig. 2, the transmitter- and receiver-side beam directions of a radio ray between TX(0, 0, 10) and RX(0, $-5$, 5) are $\overrightarrow{v_t}=(4, 5, -1)$ and $\overrightarrow{v_r}=(-4, -5, -1)$, respectively. We assume that an RAT-based scatterer localization system knows the ray is reflected twice by the two surfaces of a dihedral, TOF is measured and the overall path length is 32.4 m. Different scatterers’ location and orientation illustrated in Fig. 2a–d induce four different trajectories (the trajectories from TX to RX with two reflections are represented as the concatenation of four coordinates separated by the hyphens):

- Trajectory A: (0, 0, 10)–(8, 10, 8)–(10, 7.5, 7.5)–(0, $$-5$$, 5);
- Trajectory B: (0, 0, 10)–(8.6, 10.75, 7.85)–(6, 2.5, 6.5)–(0, $$-5$$, 5);
- Trajectory C: (0, 0, 10)–(5.8, 7.25, 8.55)–(10.9, 8.62, 7.73)–(0, $$-5$$, 5);
- Trajectory D: (0, 0, 10)–(0.8, 1, 9.8)–(11.1, 8.88, 7.78)–(0, $$-5$$, 5).

It is difficult to distinguish the true trajectory from trajectories A to D by conventional scatterer localization methods because trajectories A–D are characterized by the same TX/RX locations, the same transmitter- and receiver-side beam direction, and the same overall path length of 32.4 m.

## Results

### Simulations

Since wood, plaster, and glass are the most common materials in indoor environments (see Fig. 3 for an exemplary indoor environment), RLs induced by these materials are studied and simulated in this article. The simulation configuration is shown in Fig. 4. A TX antenna and an RX antenna are placed in an environment with the scatterers. By transmitting a highly directional radio ray from the TX, a single- and a double-bounce reflection trajectory between the TX and the RX can be simulated as shown in Fig. 4a, b, respectively. The vertical walls can be made of one of the three common building materials in indoor environments, namely wood, plaster, and glass. The materials are homogeneous, and the parameters of the materials in the simulation recommended by the International Telecommunications Union (ITU) [12] are given in Table 1. For single-bounce reflection, a radio ray transmitted by the TX strikes the vertical wall with an incident angle $\theta _i$ as shown in Fig. 4a [13], $\theta _i$ is swept from 0$^\circ$ to 80$^\circ$ by changing the position of the RX. $d_t$ and $d_r$ are the path lengths between the RP and the TX/RX, respectively. *d* is the total path length and $d=d_t+d_r$. For double-bounce reflection, a radio ray transmitted by the TX is reflected by the two surfaces of the dihedral at incident angles $\theta _{i1}$ and $\theta _{i2}$ as illustrated in Fig. 4b. According to ray tracing and geometry theory, the angle between two dihedral surfaces $\theta$ is equal to $\theta _{i1}$ plus $\theta _{i2}$. By steering transmitter-side beam direction and changing dihedral angle $\theta$, the combinations of ($\theta _{i1}, \theta _{i2}$) with any $\theta _{i1}$ and $\theta _{i2}$ value can be simulated. Let $d_1$, $d_2$, and $d_3$ be the distances between the TX and the RP1, the RP1 and the RP2, and the RP2 and the RX, respectively. Then, the total path length *d* of this double-bounce trajectory is equal to $d_1+d_2+d_3$.

**Table 1:** Parameters of three common building materials recommended by ITU-R P.2040-2

Material	Permittivity		Conductivity	
	*a*	*b*	*c*	*d*
Wood	1.99	0	0.0047	1.0718
Plaster	2.73	0	0.0085	0.9395
Glass	6.31	0	0.0036	1.3394

**Table 2:** A database of RLs induced by wood, plaster, and glass at 100 GHz

Material	RL in decibels at different incident angles in degrees																
	0$^\circ$	5$^\circ$	10$^\circ$	15$^\circ$	20$^\circ$	25$^\circ$	30$^\circ$	35$^\circ$	40$^\circ$	45$^\circ$	50$^\circ$	55$^\circ$	60$^\circ$	65$^\circ$	70$^\circ$	75$^\circ$	80$^\circ$
Wood	16.42	16.41	16.38	16.33	16.25	16.13	15.95	15.68	15.28	14.7	13.9	12.86	11.55	10.01	8.26	6.36	4.34
Plaster	12.44	12.44	12.43	12.42	12.39	12.34	12.25	12.11	11.89	11.55	11.07	10.4	9.52	8.42	7.1	5.59	3.9
Glass	7.31	7.31	7.31	7.31	7.31	7.30	7.28	7.25	7.2	7.12	7	6.81	6.54	6.14	5.56	4.75	3.63

**Table 3:** Information about possible trajectories and scatterers after matching the overall path length

Trajectory	RP1	RP2	$\theta _{i1}$	$\theta _{i2}$	Scatterer1	Scatterer2	RL1 (dB)	RL2 (dB)	$\Sigma$RL
A	(8, 10, 8)	(10, 7.5, 7.5)	39.5$^\circ$	51.9$^\circ$	Wood	Wood	15.32	13.54	28.86
A	(8, 10, 8)	(10, 7.5, 7.5)	39.5$^\circ$	51.9$^\circ$	Plaster	Plaster	11.91	10.84	22.75
A	(8, 10, 8)	(10, 7.5, 7.5)	39.5$^\circ$	51.9$^\circ$	Glass	Glass	7.2	6.93	14.13
A	(8, 10, 8)	(10, 7.5, 7.5)	39.5$^\circ$	51.9$^\circ$	Wood	Plaster	15.32	10.84	26.16
A	(8, 10, 8)	(10, 7.5, 7.5)	39.5$^\circ$	51.9$^\circ$	Plaster	Wood	11.91	13.54	25.45
A	(8, 10, 8)	(10, 7.5, 7.5)	39.5$^\circ$	51.9$^\circ$	Wood	Glass	15.32	6.93	22.25
A	(8, 10, 8)	(10, 7.5, 7.5)	39.5$^\circ$	51.9$^\circ$	Glass	Wood	7.2	13.54	20.74
A	(8, 10, 8)	(10, 7.5, 7.5)	39.5$^\circ$	51.9$^\circ$	Plaster	Glass	11.91	6.93	18.84
A	(8, 10, 8)	(10, 7.5, 7.5)	39.5$^\circ$	51.9$^\circ$	Glass	Plaster	7.2	10.84	18.04
B	(8.6, 10.75, 7.85)	(6, 2.5, 6.5)	13.8$^\circ$	79.6$^\circ$	Wood	Wood	16.35	4.51	20.86
B	(8.6, 10.75, 7.85)	(6, 2.5, 6.5)	13.8$^\circ$	79.6$^\circ$	Plaster	Plaster	12.42	4.05	16.47
B	(8.6, 10.75, 7.85)	(6, 2.5, 6.5)	13.8$^\circ$	79.6$^\circ$	Glass	Glass	7.31	3.73	11.04
B	(8.6, 10.75, 7.85)	(6, 2.5, 6.5)	13.8$^\circ$	79.6$^\circ$	Wood	Plaster	16.35	4.05	20.4
B	(8.6, 10.75, 7.85)	(6, 2.5, 6.5)	13.8$^\circ$	79.6$^\circ$	Plaster	Wood	12.42	4.51	16.93
B	(8.6, 10.75, 7.85)	(6, 2.5, 6.5)	13.8$^\circ$	79.6$^\circ$	Wood	Glass	16.35	3.73	20.08
B	(8.6, 10.75, 7.85)	(6, 2.5, 6.5)	13.8$^\circ$	79.6$^\circ$	Glass	Wood	7.31	4.51	11.82
B	(8.6, 10.75, 7.85)	(6, 2.5, 6.5)	13.8$^\circ$	79.6$^\circ$	Plaster	Glass	12.42	3.73	16.15
B	(8.6, 10.75, 7.85)	(6, 2.5, 6.5)	13.8$^\circ$	79.6$^\circ$	Glass	Plaster	7.31	4.05	11.36
C	(5.8, 7.25, 8.55)	(10.9, 8.62, 7.73)	72.1$^\circ$	20.1$^\circ$	Wood	Wood	7.48	16.25	23.73
C	(5.8, 7.25, 8.55)	(10.9, 8.62, 7.73)	72.1$^\circ$	20.1$^\circ$	Plaster	Plaster	6.49	12.39	18.88
C	(5.8, 7.25, 8.55)	(10.9, 8.62, 7.73)	72.1$^\circ$	20.1$^\circ$	Glass	Glass	5.26	7.31	12.57
C	(5.8, 7.25, 8.55)	(10.9, 8.62, 7.73)	72.1$^\circ$	20.1$^\circ$	Wood	Plaster	7.48	12.39	19.87
C	(5.8, 7.25, 8.55)	(10.9, 8.62, 7.73)	72.1$^\circ$	20.1$^\circ$	Plaster	Wood	6.49	16.25	22.74
C	(5.8, 7.25, 8.55)	(10.9, 8.62, 7.73)	72.1$^\circ$	20.1$^\circ$	Wood	Glass	7.48	7.31	14.79
C	(5.8, 7.25, 8.55)	(10.9, 8.62, 7.73)	72.1$^\circ$	20.1$^\circ$	Glass	Wood	5.26	16.25	21.51
C	(5.8, 7.25, 8.55)	(10.9, 8.62, 7.73)	72.1$^\circ$	20.1$^\circ$	Plaster	Glass	6.49	7.31	13.8
C	(5.8, 7.25, 8.55)	(10.9, 8.62, 7.73)	72.1$^\circ$	20.1$^\circ$	Glass	Plaster	5.26	12.39	17.65
D	(0.8, 1, 9.8)	(11.1, 8.88, 7.78)	83.1$^\circ$	11.3$^\circ$	Wood	Wood	3.06	16.37	19.43
D	(0.8, 1, 9.8)	(11.1, 8.88, 7.78)	83.1$^\circ$	11.3$^\circ$	Plaster	Plaster	2.77	12.43	15.2
D	(0.8, 1, 9.8)	(11.1, 8.88, 7.78)	83.1$^\circ$	11.3$^\circ$	Glass	Glass	2.73	7.31	10.04
D	(0.8, 1, 9.8)	(11.1, 8.88, 7.78)	83.1$^\circ$	11.3$^\circ$	Wood	Plaster	3.06	12.43	15.49
D	(0.8, 1, 9.8)	(11.1, 8.88, 7.78)	83.1$^\circ$	11.3$^\circ$	Plaster	Wood	2.77	16.37	19.14
D	(0.8, 1, 9.8)	(11.1, 8.88, 7.78)	83.1$^\circ$	11.3$^\circ$	Wood	Glass	3.06	7.31	10.37
D	(0.8, 1, 9.8)	(11.1, 8.88, 7.78)	83.1$^\circ$	11.3$^\circ$	Glass	Wood	2.73	16.37	19.1
D	(0.8, 1, 9.8)	(11.1, 8.88, 7.78)	83.1$^\circ$	11.3$^\circ$	Plaster	Glass	2.77	7.31	10.08
D	(0.8, 1, 9.8)	(11.1, 8.88, 7.78)	83.1$^\circ$	11.3$^\circ$	Glass	Plaster	2.73	12.43	15.16

**Table 4:** Information about possible trajectories and scatterers after matching the measured RL

Trajectory	RP1	RP2	$\theta _{i1}$	$\theta _{i2}$	Scatterer1	Scatterer2	Path length (m)
1	(12, 15, 17)	(17, 16.25, 9.25)	65.3$^\circ$	19.3$^\circ$	Wood	Wood	57.3
2	(5, 6.25, 8.75)	(9, 6.25, 7.25)	64.6$^\circ$	29.2$^\circ$	Wood	Wood	27
3	(13, 16.25, 6.75)	(17, 16.25, 9.25)	57$^\circ$	26.4$^\circ$	Plaster	Wood	53.3
4	(7, 8.75, 8.25)	(10, 7.5, 7.5)	53.2$^\circ$	37.1$^\circ$	Plaster	Wood	30.8
5	(12, 15, 7)	(15, 13.75, 8.75)	48.9$^\circ$	35.9$^\circ$	Wood	Plaster	47.4
6	(8, 10, 8)	(10, 7.5, 7.5)	39.5$^\circ$	51.9$^\circ$	Wood	Plaster	32.4
7	(11, 13.75, 7.25)	(13, 11.25, 8.25)	37.5$^\circ$	48.5$^\circ$	Plaster	Wood	42.2
8	(15, 18.75, 6.25)	(16, 15, 9)	27.7$^\circ$	56.8$^\circ$	Wood	Plaster	55
9	(15, 18.75, 6.25)	(15, 13.75, 8.75)	20$^\circ$	65.2$^\circ$	Wood	Wood	54.9

**Table 5:** Possible materials of RP1-RP3 derived from two independent trajectories

Trajectory	Possible materials of RP1–RP3		
	RP1	RP2	RP3
Trajectory 1	Plaster	Glass	
Trajectory 1	Glass	Plaster	
Trajectory 2	Wood		Plaster
Trajectory 2	Glass		Wood

By the simulation steps proposed in [13], RL of single- and double-bounce reflection can be obtained. The received power at the RX side, which is attenuated by free-space propagation and reflection at a certain incident angle, is measured in the first step. The overall path loss (PL) in decibels including RL and free-space path loss (FSPL) of the trajectory is calculated as

1 $$\begin{aligned} \text{PL} = P_{\text{TX}} - P_{\text{RX}}, \end{aligned}$$

where $P_{\text{TX}}$ is the transmit power in dBm and $P_{\text{RX}}$ is the received power in dBm.

In the second step, the RX is placed at the distance *d* ($d=d_t+d_r$ for single-bounce or $d=d_1+d_2+d_3$ for double-bounce) from the TX. The TX transmits the same radio ray as the one in step 1. Therefore, a LOS trajectory between the TX and the RX is established as illustrated in Fig. 4c. The FSPL at a distance of *d* can be measured, or can be calculated by Friis’ equation:

2 $$\begin{aligned} \text{FSPL}(f,d) = 32.4 +20\log _{10}(f) + 20\log _{10}(d), \end{aligned}$$

where *f* is the carrier frequency in Mega Hertz and *d* is the path length in kilometers.

Finally, the RL can be calculated by subtracting (2) from (1):

3 $$\begin{aligned} \text{RL} = \text{PL} - \text{FSPL}(f,d). \end{aligned}$$

It is worth noting that RL takes no account of loss in free space prior to or subsequent to the interaction of a wave with the scatterer [12]; RL depends on the interaction between the wave and the scatterer only. Antenna type, antenna gain, transmit power, and path length do not affect RL. A change in these factors brings a corresponding change in RSS. For example, if the $P_{\text{TX}}$ increases by 3 dB, the measured RSS increases by 3 dB accordingly. From (1) to (3), the increased transmit power has no impact on PL and RL at all.

The RL simulation results of single-bounce reflection induced by scatterers made of wood, plaster, and glass at different incident angles $\theta _i$ at 100 GHz are reported in Table 2 and Fig. 5a. The roughness of wood, plaster, and glass in the simulation is 0.4 mm, 0.2 mm, and 0 mm (perfectly smooth), respectively. The roughness represents the root-mean-square value of the height deviation from a perfectly smooth surface [14]. From Fig. 5a, for scatterers made of specific material, RL and incident angle are negatively correlated, and a large incident angle induces a small RL [13].

The sum of reflection losses ($\Sigma$RL) simulation results of double-bounce reflection induced by two scatterers made of any combinations of wood, plaster, and glass at incident angles $\theta _{i1}$ and $\theta _{i2}$ at 100 GHz is reported in Fig. 5b. In Fig. 5b, *x*-axis represents the first incident angle $\theta _{i1}$ of a radio ray reflected by the first scatterer, *y*-axis represents the second incident angle $\theta _{i2}$ of the same ray reflected by the second scatterer, and *z*-axis represents the $\Sigma$RL (RL1 plus RL2) induced by the two scatterers. The collection of $\Sigma$RL data of each sequence-of-material is plotted to a surface and displayed with specific color as shown in Fig. 5b. A sequence-of-material, which contains a set of materials, represents the material of scatterers in temporal order when the radio ray strikes the scatterers in sequence. The legends in Fig. 5b show all sequences-of-material of double-bounce reflection. For example, the magenta surface in Fig. 5b represents the $\Sigma$RL induced by double-bounce reflection, the first scatterer is made of wood, and the second scatterer is made of plaster. From Fig. 5b, it is evident that the $\Sigma$RLs induced by different sequences-of-material are significantly different, especially when both incident angles $\theta _{i1}$ and $\theta _{i2}$ are small. When both incident angles increase, a sharp reduction in $\Sigma$RL is observed. This is expected behavior because the $\Sigma$RL of double-bounce reflection can be divided into two single-bounce reflections induced by two scatterers, respectively, and each single-bounce reflection induces low RL at large incident angle as shown in Fig. 5a.

From the simulation results, it is worth noting that the $\Sigma$RL induced by two scatterers is independent of the scatterers’ sequence-of-material when a radio ray strikes the two scatterers. For example, a radio ray reflected by a woody surface at incident angle $\theta _{i1}$ of 50$^\circ$ first and then reflected by a glassy surface at incident angle $\theta _{i2}$ of 75$^\circ$ has same $\Sigma$RL of 18.65 dB as the same radio ray reflected by a glassy surface at incident angle $\theta _{i1}$ of 75$^\circ$ first and then reflected by a woody surface at incident angle $\theta _{i2}$ of 50$^\circ$. Therefore, by $\Sigma$RL of double-bounce reflection, the scatterers’ material combination may be concluded, but the sequence-of-material of the two scatterers is still uncertain.

### Theory

The RL simulation results in Fig. 5 can be derived from Fresnel equations [12] as follows. The relative permittivity of a material is given by

4 $$\begin{aligned} \eta = af^b -j17.98cf^d/f, \end{aligned}$$

where $\eta$ is the relative permittivity of the material; *a*, *b*, *c*, and *d* are material properties that determine the relative permittivity and conductivity. The values of *a*, *b*, *c*, and *d* are given in Table 1.

Fresnel reflection coefficients $r_{\text{TE}}$ and $r_{\text{TM}}$ for transverse electric (TE) and transverse magnetic (TM) polarization describe the ratio of the amplitude of the reflected wave to the amplitude of the incident wave when the wave incidents upon a material at a certain frequency and at incident angle $\theta _i$ from the air [12]:

5 $$\begin{aligned} r_{\text{TE}}= & {} \frac{\text{cos}{\theta _i}-\sqrt{\eta -\text{sin}^2{\theta _i}}}{\text{cos}{\theta _i}+\sqrt{\eta -\text{sin}^2{\theta _i}}}, \end{aligned}$$

6 $$\begin{aligned} r_{\text{TM}}= & {} \frac{{\eta }\text{cos}{\theta _i}-\sqrt{\eta -\text{sin}^2{\theta _i}}}{{\eta }\text{cos}{\theta _i}+\sqrt{\eta -\text{sin}^2{\theta _i}}}. \end{aligned}$$

As explained above, RL is dependent on the material of the scatterer, radio frequency, and incident angle only. This conclusion is also well supported by Fresnel equations (4)–(6). From (4) to (6), it is clear that reflection coefficients take no account of free-space losses, but only the effect of the media interface [12]. Antenna type, antenna gain, transmit power, and path length do not contribute to RL at all.

Figure 6 shows the reflection coefficient amplitude for a wave at 100 GHz in the air striking wood, plaster, and glass over a range of incident angles from 0$^\circ$ to 90$^\circ$ for both TE and TM polarization using (4) to (6) and taking the properties of materials in Table 1. But in practice, we are more interested in formulae that determine reflection coefficient power, since the power of radio signals can be directly measured by radio transceivers. The power of a wave is generally proportional to the square of the wave’s amplitude. Therefore, Fresnel reflection coefficient power $R_{\text{TE}}$ and $R_{\text{TM}}$ are the square of $r_{\text{TE}}$ and $r_{\text{TM}}$:

7 $$\begin{aligned} R_{\text{TE}}= & {} |r_{\text{TE}}|^2, \end{aligned}$$

8 $$\begin{aligned} R_{\text{TM}}= & {} |r_{\text{TM}}|^2. \end{aligned}$$

In a JCAS network, we assume that the cross-polarized antennas can be corrected through calibration, to achieve an average power coefficient $R_{\text{av}}$ for both polarizations:

9 $$\begin{aligned} R_{\text{av}} = \frac{1}{2}(R_{\text{TE}}+R_{\text{TM}}). \end{aligned}$$

Figure 7 shows the reflection coefficient power for a wave at 100 GHz in the air striking wood, plaster, and glass over a range of incident angles from 0$^\circ$ to 90$^\circ$ for both TE and TM polarization using (4) to (9) and taking the properties of materials in Table 1.

Fresnel equations (4)–(9) can be applied to perfectly smooth surfaces only. In order to include scattering loss induced by rough surface, the Fresnel reflection coefficients $r_{\text{TE}}$ and $r_{\text{TM}}$ should be multiplied by the Rayleigh roughness factor $\rho$ [15]:

10 $$\begin{aligned} {r'_{\text{TE}}}= & {} \rho \cdot {r_{\text{TE}}}, \end{aligned}$$

11 $$\begin{aligned} {r'_{\text{TM}}}= & {} \rho \cdot {r_{\text{TM}}}, \end{aligned}$$

where $r'_{\text{TE}}$ and $r'_{\text{TM}}$ are the modified reflection coefficients with the impact of roughness for TE and TM polarization, respectively. The Rayleigh roughness factor $\rho$ is given by

12 $$\begin{aligned} \rho = \text{e}^{-\frac{g}{2}}, \end{aligned}$$

with

13 $$\begin{aligned} g = \left( \frac{4\pi {\sigma }_R\text{cos}{\theta _i}}{\lambda }\right) ^2, \end{aligned}$$

where *g* is a parameter established for the effect of roughness [16], $\sigma _R$ is the standard deviation of the surface roughness, and $\lambda$ is the wavelength of the radio wave.

A summary of the RL calculation consists of the following steps. First, perform relative permittivity calculation by using (4) and taking the parameters of the materials from Table 1. Second, the TE and TM components of a radio wave can be separately calculated by using (5)–(8), then they are combined by using (9). The impact of roughness on RL can be involved by (10)–(13). Finally, the RL can be calculated by converting the effective power coefficient $R_{\text{av}}$ in percentage terms into RL in decibels.

## Methods

To tackle the challenges of RAT-based scatterer localization and material identification in the rich scattering environment, we propose two novel methods all based on RL to localize scatterers and identify scatterers’ materials simultaneously for NLOS trajectories. These methods do not require any prior knowledge of the environment.

### Method 1

The first method (referred to as “method 1” below) comprises the following steps:

- Step 1: calculate RL or $\Sigma$RL induced by scatterers under different scenarios at various frequencies to be used for scatterer localization. A specific scenario consists of the following information: the material and incident angle of the *n*th scatterer ($n=1, 2, \ldots , N$). The scenario information (materials and incident angles), frequencies, and corresponding RLs are stored in an RL database.
- Step 2: establish an NLOS trajectory between TX and RX in an environment to be detected at a certain frequency and measure the RL or $\Sigma$RL for this trajectory by using the measurement method proposed in Sect. 3.
- Step 3: identify a set of possible trajectories by matching the overall path length.
- Step 4: for the possible trajectories obtained by Step 3, identify the true trajectory by comparing the measured RL or $\Sigma$RL at a certain frequency with pre-calculated RL or $\Sigma$RL in the RL database created in Step 1.

For the sake of simplicity, we assume that all scatterers in an environment are made of the materials listed in Table 1 only, and the number of bounces of each trajectory is no more than two. Hence, the NLOS trajectories are divided into two categories: single-bounce reflection ($N=1$) with one RP and double-bounce reflection ($N=2$) with two RPs. For single-bounce reflection, the scatterer can be made of any of the three materials, namely wood, plaster, and glass. For double-bounce reflection, there are three possibilities of material for the first scatterer, and three for the second scatterer. Therefore, there are nine possible sequences-of-material of the two scatterers in total. In Step 1, an RL data collecting phase is needed before the utilization of the proposed method. RLs induced by any sequences-of-material at any incident angles are calculated by Fresnel equations, then all the relevant information is stored in an RL database. An example of an RL database of single-bounce reflection is shown in Table 2. By using the information in Table 2, the single-bounce RL induced by any material at any incident angle can be obtained. For multiple-bounce reflection, the $\Sigma$RL can be obtained by adding the RL of each reflection together.

We take the scenario in Fig. 8 as an example to illustrate the proposed method 1. In Step 2, a radio ray transmitted from the TX at T(0, 0, 10) in the direction of vector $\overrightarrow{v_t}=(4, 5, -1)$ is reflected by unknown scatterers (i.e., unknown position, unknown number of reflection bounces, and unknown material of scatterers), and the same ray is received by the RX at R(0, $-5$, 5) in the direction of vector $\overrightarrow{v_r}=(-4, -5, -1)$. We assume that the overall path length is 32.4 m according to TOF measurement, and measured RL or $\Sigma$RL is 22.25 dB at 100 GHz by using the measurement method proposed in Sect. 3. The goal is to trace the trajectory, localize the RPs, and identify the material of scatterers for this radio ray.

Vector $\overrightarrow{v_t}=(4, 5, -1)$ and vector $\overrightarrow{v_r}=(-4, -5, -1)$ are not co-planar; in other words, the transmitter- and receiver-side beam cannot intersect at a point. Therefore, this radio ray is impossible to be a trajectory with single-bounce reflection. In Step 3, we first determine a set of possible trajectories by matching the overall path length *d* ($d=c\cdot {t}$ where *c* is the speed of light and *t* is TOF). The aim is to localize a pair-of-RPs lying on this double-bounce reflection trajectory. To achieve this, we distribute evenly spaced points on the transmitter- and receiver-side-beam ray, respectively. As shown in Fig. 8, a collection of points lying on the transmitter-side-beam ray is defined as set $P=\{P_1, P_2, \ldots , P_i, \ldots \}$, the distance between any adjacent points is $\Delta d$. The selection of $\Delta d$ needs to carefully balance localization accuracy and computational complexity; a shorter $\Delta d$ can improve localization accuracy but leads to a relatively long latency and high computational complexity. The coordinate of any point in set *P* can be obtained by the coordinate of the initial point (0, 0, 10), $\Delta d$, the transmitter-side beam direction $\overrightarrow{v_t}$, and index *i*. Similarly, we define a set $Q=\{Q_1, Q_2, \ldots , Q_j, \ldots \}$ for receiver-side-beam ray. Then, a path-length-matching algorithm is used to identify all possible pairs-of-RPs and all possible trajectories. The algorithm consists of the following procedure:

For any point $P_i$ in set *P* and any point $Q_j$ in set *Q*, we calculate the overall double-bounce reflection path length $d(P_i,Q_j)$. $d(P_i,Q_j)$ is the sum of the lengths of the three line segments $\overline{TP_i}$, $\overline{{P_i}{Q_j}}$, and $\overline{Q_jR}$. In case of $d(P_i,Q_j) \ne c\cdot {t}$, we ignore this $(P_i,Q_j)$. Repeat this procedure until $d(P_i,Q_j)$ is longer than or equal to the overall path length of 32.4 m. Finally, four pairs-of-RPs are obtained after the algorithm enumerates all combinations of $P_i$ and $Q_j$:

- Pair A: RP1(8, 10, 8), RP2(10, 7.5, 7.5);
- Pair B: RP1(8.6, 10.75, 7.85), RP2(6, 2.5, 6.5);
- Pair C: RP1(5.8, 7.25, 8.55), RP2(10.9, 8.62, 7.73);
- Pair D: RP1(0.8, 1, 9.8), RP2(11.1, 8.88, 7.78).

The four trajectories A–D with double-bounce reflection induced by scatterers located at pairs-of-RPs A–D are depicted in Fig. 2a–d; the corresponding incident angles at each RP can be obtained by (14) and depicted in Fig. 2a–d also.

14 $$\begin{aligned} \theta = \frac{1}{2}\text{arccos}\left( \frac{u\cdot {v}}{\Vert u\Vert \Vert v\Vert }\right) , \end{aligned}$$

where *u* and *v* are the two Euclidean vectors initialed from RP and along with incident ray and outgoing ray, respectively.

In Step 4, the true pair-of-RPs is identified by comparing the measured $\Sigma$RL with the data in the RL database created in Step 1. RL induced by the first scatterer (RL1) at incident angle $\theta _{i1}$, RL induced by the second scatterer (RL2) at incident angle $\theta _{i2}$ of trajectories A-D are summarized in Table 3. From the $\Sigma$RL data in Table 3, it is evident that there are significant differences between the most of RLs of trajectories A-D with different sequences-of-material. As mentioned earlier, an RL value of 22.25 dB has already been measured at 100 GHz in Step 2. By looking up Table 3, a row with $\Sigma$RL = 22.25 dB is found. From the information contained in this row, we can conclude that trajectory A is the true trajectory, RP1 locates at (8, 10, 8) and the first scatterer is made of wood, RP2 locates at (10, 7.5, 7.5) and the second scatterer is made of glass. The information of the two RPs can be used to represent two points in 3D space with four-dimensional information including Cartesian coordinate (*x*, *y*, *z*) and material information. By collecting enough points in the environment and converting the point cloud to 3D surfaces, a 3D digital map with material information can be generated.

### Method 2

An alternative method (referred to as method 2 below) for scatterer localization and material identification can be developed by executing Steps 3 and 4 of method 1 in reverse order. Similar to method 1, method 2 carries out a four-step procedure:

- Steps 1 and 2: essentially the same as method 1.
- Step 3: find all sequences-of-material and incident angles from the RL database such that the RL induced by scatterers with these sequences-of-material and incident angles is close to measured RL.
- Step 4: estimate the positions of RPs of every possible trajectory by matching the overall path length.

Steps 1 and 2 of method 2 just do the same as the steps of method 1, namely to create an RL database and then measure the RL of a trajectory in the environment to be detected. In Step 3, RL matching is performed; in other words, each RL in the RL database is compared with the measured RL, and the rows with the same RL as measured RL are identified. The purpose of RL matching is to find all possible sequences-of-material and incident angles that induce the same RL as measured RL. To help visualize the possible sequences-of-material and their incident angles, the trace of the RL data surfaces in Fig. 5b is plotted. The trace of a surface is the cross section of the surface with a plane parallel to one of the coordinate planes. For example, we assume that the measured RL is 26.16 dB in the scenario depicted in Fig. 8. As shown in Fig. 9a, by intersecting plane *z* = 26.16 with the double-bounce RL data surfaces, we find three traces. Removing the RL data surfaces helps us visualize the three traces, and they are illustrated in Fig. 9b. These three traces consist of all sequences-of-material and incident angles information about trajectories that can induce RL of 26.16 dB. For example, in Fig. 9b, point (39.5, 51.9, 26.16) lying on the trace curve colored with magenta indicates that a double-bounce-reflection trajectory may contain the following propagation properties: (1) the first reflection is induced by a scatterer made of wood at incident angle $\theta _{i1}$ of 39.5$^\circ$; (2) the second reflection is induced by a scatterer made of plaster at incident angle $\theta _{i2}$ of 51.9$^\circ$; (3) the $\Sigma$RL of this trajectory is 26.16 dB. By Step 3, we can find all possible sequences-of-material and incident angles of double-bounce reflection with RL of 26.16 dB.

In Step 4, we first calculate the incident angles of all possible trajectories using AOD, AOA, and coordinates of TX and RX. Similar to Step 3 of method 1, as shown in Fig. 8, for any point $P_i$ in set *P* and any point $Q_j$ in set *Q*, joining T(0, 0, 10), $P_i$, $Q_j$, and R(0, -5, 5) in sequence can trace a trajectory. The first incident angle $\theta _{i1}$ ($\theta _{i1}=\widehat{TP_i{Q_j}}/2$) and the second incident angle $\theta _{i2}$ ($\theta _{i2}=\widehat{P_i{Q_j}R}/2$) of this trajectory can be obtained by using (14). In Fig. 10, we show the incident angles $\theta _{i1}$ and $\theta _{i2}$ of every possible trajectory by enumerating all combinations of $P_i$ and $Q_j$ until the trajectory length is longer than 32.4 m. The incident angles of each trajectory are marked by star (*); the *y*- and *x*-coordinates of a star marker represent the first incident angle $\theta _{i1}$ and the second incident angle $\theta _{i2}$ of a trajectory, respectively. If we combine the three traces in Fig. 9b into Fig. 10, the traces intersect the black star markers at several points $T_1, T_2, \ldots , T_9$. These points represent nine trajectories 1–9 that satisfy the following conditions: (1) the RPs induce $\Sigma$RL of 26.16 dB; (2) the RPs lie on the transmitter- and the receiver-side beam ray, respectively. Table 4 summarizes the coordinates of RPs, incident angles, and sequence-of-material of trajectories 1–9. The overall path lengths of trajectories 1–9 are also calculated by coordinates of RPs. Finally, by comparing the path length of trajectories 1–9 with the measured path length of 32.4 m, it can be concluded that trajectory 6 is the true trajectory.

## Discussion

The proposed methods localize scatterers by RL which is obtained by measured RSS. Therefore, their performance is highly affected by the accuracy of RSS measurement (RSS uncertainty) [4]. RSS uncertainty may result from non-ideal equipment, hardware impairment, Gaussian random noise, and interference from neighboring antennas in a JCAS network. To reduce RSS uncertainty, we can perform multiple RSS measurements, then calculate the mean RSS, and subsequently estimate the RL to be used in the proposed methods [17]. With the help of multiple measurements, a relatively long measurement period can achieve better SNR and lower standard error of RSS uncertainty than RSS uncertainty by a single measurement. The RSS-Measurement-Muting (RMM) can be used to mitigate interference from the environment for RSS measurement also. RMM delays or avoids the transmission of any messages of neighboring nodes for a time period when an RSS measurement is performed by a JCAS system.

Another strategy to reduce the uncertainty of material estimation is inter-trajectory data fusion. Because the material data derived from different trajectories are uncorrelated, by integrating these uncorrelated data, material estimation results with less uncertainty can be obtained. The example in Fig. 11 demonstrates the benefit of inter-trajectory data fusion to reduce the uncertainty of material estimation [18]. In a two-story building, two double-bounce-reflection trajectories TX1$\rightarrow$RP1$\rightarrow$RP2$\rightarrow$RX and TX2$\rightarrow$RP1$\rightarrow$RP3$\rightarrow$RX (referred to as “trajectory 1” and “trajectory 2” below) are created between TX1-RX and TX2-RX, respectively. By using the material identification method, the materials of RP1 and RP2 derived from RL of trajectory 1 and the materials of RP1 and RP3 derived from RL of trajectory 2 are given in Table 5. The material of the reflecting surface at each RP has two possibilities. Therefore, the materials of these RPs cannot be determined when the trajectory data are used individually. However, combining the material data from two trajectories can yield the material of RP1 (glass) because glass is the common possible material derived from both trajectories. The materials of RP2 and RP3 can be concluded consequently.

## Conclusions

Reusing radio signals of cellular networks to localize scatterers will potentially replace some functionalities of radar/LiDAR in 6G, for example, 3D mapping. However, there has been little quantitative research on this topic so far. The process of scatterer localization can be simplified by making single-bounce-assumption as a workaround, but it cannot meet the extreme reliability requirements of some critical use cases, e.g., AD. In this article, we extend the capability of the method proposed in [13] from material identification only to both material identification and scatterer localization simultaneously. The extension is based on the findings from RL simulations and theoretical analysis: (1) most of the $\Sigma$RLs induced by different sequences-of-material are significantly different; (2) RL is dependent on the material of the scatterer, radio frequency, and incident angle only; (3) $\Sigma$RLs induced by multiple scatterers are independent of scatterers’ sequence-of-material. By extracting material and incident angle information from RL, the RAT-based scatterer localization and material identification systems can be developed. The proposed methods will be able to support passive localization cost-effectively by eliminating the need for dedicated hardware (e.g., sensor) and external assistance (e.g., 3D digital map database) by using the only cellular infrastructure.

## Data Availability

The datasets used and/or analyzed during the current study are available from the corresponding author on reasonable request.

## Abbreviations

RAT: Radio access technology
JCAS: Joint communication and sensing
RL: Reflection loss
UE: User equipment
VR: Virtual reality
3D: Three-dimensional
AD: Autonomous driving
AOD: Angle of departure
AOA: Angle of arrival
TOF: Time of flight
RSS: Received signal strength
NLOS: Non-line of sight
LiDAR: Light detection and ranging
RP: Reflection point
ITU: International Telecommunications Union
PL: Path loss
FSPL: Free-space path loss
TE: Transverse electric
TM: Transverse magnetic
RMM: RSS measurement muting
